# Nutrition education improves knowledge and BMI-for-age in Ghanaian school-aged children

**DOI:** 10.4314/ahs.v21i2.55

**Published:** 2021-06

**Authors:** Reginald A Annan, Charles Apprey, Godwin O Agyemang, Diane M Tuekpe, Odeafo Asamoah-Boakye, Satoru Okonogi, Taro Yamauchi, Takeshi Sakurai

**Affiliations:** 1,2 Department of Biochemistry and Biotechnology, Faculty of Biosciences, College of Science, Kwame Nkrumah University of Science and Technology, Kumasi, Ghana; 3 Department of Agricultural and Resource Economics, School of Agricultural and Life Sciences, University of Tokyo, Japan; 4 Department of Health Sciences, School of Medicine, Hokkaido University, Japan

**Keywords:** School-aged children, nutrition education, BMI-for-age, nutrition knowledge, basic school

## Abstract

**Background:**

Adequate nutrition is required for growth and development in children. This study tested the effectiveness of nutrition education on knowledge and BMI-for-age (BFA) of school-aged children in the Kumasi Metropolis.

**Methods:**

Children, aged 9–13 years old were recruited from ten randomly selected primary schools in the Metropolis. The schools were randomly allocated into 3 groups: nutrition education (3 schools), physical activity (PA) education (3 schools), both interventions (2 schools), or control (2 schools). Following a baseline nutrition and PA knowledge and status assessment in 433 children, twice-monthly nutrition and PA education and demonstrations were carried out for 6 months, followed by a post-intervention assessment.

**Results:**

PA and nutrition knowledge improved in all groups (P<0.001); the highest improvement was among those who received both interventions (31.0%), followed by the nutrition education group (29.8%), and the least, the control group (19.1%). Overall, BFA improved by +0.36, from baseline (-0.26) to end of the intervention (+0.10, P<0.001). Within the groups, the nutrition group (+0.65, P<.001) had the highest improvement, then, both the intervention group (+0.27, P<0.001), the PA group (+0.23, P<0.001) and lastly, the control group (+0.18, P=0.001).

**Conclusion:**

Nutrition education could improve knowledge and BMI-for-age in school-aged children in Ghana.

## Introduction

The 2014 Ghana Demographic and Health Survey reported that 19% of Ghanaian children under five were too short for their age, ie stunted (below -2 SD) and 5% were severely stunted (below -3 SD), a decrease from the figures of 28 percent and 10%, respectively, in 2008.^1^ Five percent of the children were wasted and less than 1 percent were severely wasted, while 11 % were underweight and 2 %severely underweight, also a decrease from the 2008 figures of 14 % and 3%, respectively1. Among 5–12 years old children in a part of Ghana, the prevalence of stunting in schools on School Feeding Programme (SFP) was 16.2% compared with 17.2% among those that did not implement the SFP.^2^ The prevalence of thinness was two times higher (9.3%) among children in schools on the SFP than in children in schools that did not implement the SFP (4.6%).2 On the other hand, the prevalence of overweight was 1.9% in SFP schools and 0.0% in non-SFP implementing schools.^2^ A study by Fentiman et al.^3^ showed that 44% of 645 school-aged children in the rural aras of the eastern part of Ghana were stunted with 70% of them being anemic. Therefore, although no national data on anthropometric of Ghanaian school-aged children exist, pockets of research in this age group suggest undernutrition is equally a problem, including poor micronutrient intake 4, just as observed in under-five children. However, the school-age is completely neglected in most national policies in Ghana.^3^

Nutrition in childhood is vital and cannot be underestimated since childhood is the stage of development where mutable health behaviors are mostly transferred to adolescence and the adult stage.^5^ Out of 10.9 million deaths each year among children in the developing countries, about 60% are linked with malnutrition^6^ and malnutrition at the childhood stage can affect brain development as well as physical growth.^7^ This has implications for the development of these children and the realization of their future adult potential. Poor health and nutritional status have been associated with inadequate dietary intake.^8^ Nutrition education is important in raising awareness for people to make the right choices for healthy eating.^9^ The United Nations Food and Agriculture Organization over the years through its Nutrition Education and Consumer Awareness team provides technical support for policies and programmes to increase public awareness of the importance of eating well, to foster healthy food choices, and build the capacities of individuals and communities to adopt food and nutrition practices to promote health.^9^

Adequate nutrition and physical activity have been shown to improve academic achievement.^10^ The school is an important arena for nutrition education to support the pupil's eating patterns.^11^ Studies on nutrition intervention programs in the school setting, primarily basic school have been widely elucidated.^12^, ^13^ Although, some studies have had positive outcomes, there are reported limitations in the implementation of the nutrition program in the school setting. In Ghana, a study by Gelli et al.^13^ analysed the effectiveness of the Ghana School Feeding Programme on a child's growth among school children aged 5–15 years old. Gelli et al. study reported that the school feeding intervention in Ghana improved in height-for-age in girls aged 5–8 years, in children aged 5–8 years in poor households, but had no overall effect on BMI-for-age in the children aged 5–15 years.^13^ A limitation to Gelli et al. study was a delay in the implementation of the school feeding programme which affected a child's daily uptake of school meals.^13^ A systematic review by Gurra et al.^14^ reported a limitation to nutrition and physical activity intervention program in schools was the lack of theoretical model used in the preparation and implementation of the programs, which created inconsistencies regarding the variables analysed in the studies. Another study by Angeles-Agdepa et al.^15^ in the Philippines found that school-based nutrition education was effective in improving weight and haemoglobin of the studied children, as well as improving nutrition knowledge, attitude, and practices of the participant's mothers. Tarro et al.^16^ also reported that school-based healthy lifestyle programmes reduced obesity prevalence in school children by -2.36 percent and improved BMI-for-age for boys in the intervention group. In the UK, a study by Lloyd et al.^12^, which used a theoretical-based model in implanting a healthy lifestyle programme among primary-school children found no significant effect on overweight and obesity prevalence but a positive change in BMI scores in the intervention group. Lloyd et al.^12^ also reported that the theoretical basis for nutrition intervention programmes in school children is laudable but the effectiveness and practicality is lacking. Other studies have reported that nutrition education intervention among school children is limited to cross-communication of nutrition and physical activity knowledge received between the intervention group the control group, and self-reporting of study variables such as dietary behaviour and physical activity level.^16^, ^17^. The inconsistencies in results/limitations of the reported studies stimulate more researches on school-based nutrition programs to be carried out in different settings, to provide more insight into the subject matter. The literature above shows that programmes that aim at testing the effectiveness of nutrition education in school-aged children is much known in the high-income countries.^12^,^16^, ^18^–^20^. Less is known about this intervention strategy in Ghana and other low-and middle-income countries.^13^, ^21^. The study aimed to test the effectiveness of nutrition education on knowledge and anthropometric (BMI-for-age) status of school-aged children in the Kumasi Metropolis.

## Materials and Methods

### Study Design

The design was a longitudinal school-based intervention study.

### Study Area

The study was carried out within the Kumasi Metropolis in the Ashanti region of Ghana. The Region's location is in the South of Ghana and is the third largest of the 16 regions of Ghana, occupying a total land surface of 24,389 km2 (9,417 sq mi) or 10.2 of the total land area of Ghana. With a population of 4.780,380, Ashanti Region is the most populous of Ghana, and Kumasi is the regional capital of the Ashanti Region. The Kumasi Metropolitan Assembly is the largest district among twenty- seven (27) districts in the Ashanti region. Kumasi Metropolis was conveniently chosen for this study to represent the population of the region and Ghana at large. The Metropolis is however an important educational center in the Ashanti Region and Ghana, and has 203 government-owned primary schools; comprising of school children from different ethnicities. Sampled participants in the Kumasi metropolis can be used to define a population of school-aged children in the Ashanti region of Ghana. The city is made up of 10 sub-metropolitan areas: Manhyia, Tafo, Suame, Asokwa, Oforikrom, Asawasi, Bantama, Kwadaso, Nhyiaso and Subin.

The list of schools within the Metropolis was received from the Metropolitan Education Officer. All the 203 government-owned primary schools qualified to be selected for the study. First, the names of all schools found at each of the ten (10) sub-metropolitan towns in the Kumasi metropolis were written on paper, folded, and put in a bowl. The first school picked from each of the ten (10) sub-metropolitan towns were selected for the study. The ten schools were further randomly balloted into four groups: 3 schools allocated to the nutrition education group, 3 schools to the physical activity education group, 2 schools allocated to the combined nutrition and physical activity education group, and 2 schools to serve as a control group. Each school was randomly selected from each sub-metropolitan group of schools by simple balloting, thus reducing any bias. In each school, all children in primary 5 were selected for the study. School-going age in Ghana is defined as a child who has attained 6 years and above and can start primary education.^22^ The study used this age limit (6 years) to select participants. However, upon sampling, study participants were between ages 9 and 13 years.

The selected schools were homogenous in many regards including the kind of children who attend such schools since they are all government-owned. It is expected that children from high socio-economic income family are more likely to attend privately-owned primary schools which offer a better-quality education compared to government-owned primary schools.^23^ Thus, the children were likely to be similar in many characteristics. This means that the type of intervention each school received was the main difference between the schools. Besides, the schools were randomly allocated to the intervention type.

### Ethics

The study was approved by the Committee on Human Research Publications and Ethics of the Kwame Nkrumah University of Science and Technology (CHRPE/AP/402/17). Approval letter was obtained from the Ashanti Regional and Metropolitan Director of Education. The heads of each participating school also approved the study and agreed on dates for the research team to visit for data collection. Study aims and protocols were first explained to all children. Participation was voluntary and children who verbally assented to participate in the study were included. All participants were given informed written consent forms and signed by parents/guardians, following CHRPE regulation, before conducting the study.

### Baseline Assessment

A baseline assessment was carried out for both intervention and control groups. Participants were assessed on their anthropometrics status, physical fitness level, physical activity, and nutrition knowledge and cognition. Data was collected by researchers and other trained research assistants. The study did not obtain baseline information on whether nutrition and physical activity education was included in their curriculum and added in their learning time tables. The study did not also enquire about the physical activity level of the participants from the school teachers. However, physical education has been introduced in basic schools by the Ghana Education Service, and most government-owned primary school in Ghana have lessons on physical education on learning timetables, but its practicality is less effective^22^. Assessment of physical activity (PA) and nutrition knowledge, Attitude and Practices

Nutrition and Physical Activity KAP were assessed using a 25-questions questionnaire adapted from the Food and Agricultural Organization (FAO) nutrition and physical activity Knowledge, Attitude and Practice (KAP).^9^ Modules of the intervention were developed from topics from the Food and Agriculture Organizations Guidelines for assessing nutrition-related knowledge, attitude, and practices. This is a reference guide and a practical tool for conducting high quality-surveys of nutrition and health-related knowledge, attitude, and practices (KAP) at the school and community level. The manual was also used in developing the questionnaire for the end of the intervention assessment.^9^

The Twenty-five questions addressed physical activity knowledge, nutrition knowledge, and general knowledge of both nutrition and physical activity. There were fifteen questions on nutrition, with topics on food groups, nutrients and their deficiencies, basic health tips, and general knowledge of nutrition. The remaining ten (10) questions were on physical activity and captured topics like physical activity, locomotive and non-locomotive skills, and general knowledge of physical fitness. All questions were well-explained to all participants. The KAP questions were administered under examination conditions with effective supervision.

### Assessment of Anthropometric (Body mass index)

Anthropometric measurements, such as weight and height were taken for each participant, according to the standard nutrition assessment protocol by WHO.^24^ The height was taken in duplicates to the nearest 0.1cm using a portable stadiometer (Seca 213, Germany) and their averages were recorded. For weight measurement, the participants were made to stand on a bathroom scale (Camry bathroom weighing scale, model: DT602, India) placed on a firm flooring and set to 0.00 kg with one foot on each side of the scale and their arms placed on their sides whilst standing still and facing forward. The weight and heights were used to calculate the BMI and using WHO anthro plus software the BMI-for-age z-scores for the children were determined. All measurements were taken by trained enumerators at the schools.

Nutrition and physical activity intervention programme A 6-month intervention programme was carried out among intervention schools from June to November 2017. Each intervention group received a specified intervention programme. No education intervention was provided to the control group. Modules of the intervention were based on developmentally appropriate, culturally relevant, fun, and participatory activities. The nutrition intervention study was developed and implemented based on the evidence from studies^25^–^27^ that nutrition education can improve knowledge and BMI status in the school setting. However, these evidences are rarely studied in the Ghanaian school setting. The guiding principle was that the provision of nutrition education can help improve nutrition knowledge and practices. An improved knowledge may cause behavioral change which would overall improve anthropometric (BMI status) outcomes. The study did not assessed practices of nutrition education although the children were encouraged to practice what was taught and modules of the intervention included monthly practical sessions which allowed participants to practice nutrition and physical activity lessons given in class.

The intervention was provided mainly through conventional health education strategies; age-appropriate interactive discussions, PowerPoint Presentations, demonstrations (picture and video), field practice, and the provision of brochures/cards and posters. Sessions were held twice a month in each participating school, ideally on two consecutive days of the first week of each month. The delivery tool was the most appropriate and effective nutrition education tool to enhance understanding and improve nutrition knowledge in the school children. The delivery tool encompassed theory and practical based knowledge, demonstrations, and culturally relevant field trips, and appropriate in the Ghanaian setting. This mode of delivery has been used by Addo et al.^25^, which combined flyers, posters with nutrition information, and practical sessions as a delivery tool to improve nutrition knowledge and lipid profile of overweight/obese school children in Accra. Each intervention session lasted approximately sixty (60) minutes, twenty (20) of which was dedicated to questions and answers session. For the physical activity education, demonstration was required, and to do this, P.E kits and sports equipment were provided to facilitate the intervention. The intervention was delivered out by four (4) undergraduate biochemistry students trained for the research and supervised by an MSc public health nutrition graduate. Facilitators were trained on all the modules of the intervention and how to effectively deliver the intervention.

### Components of nutrition education

Participants in this group were given nutrition education using food groups, nutrients and their deficiencies, healthy eating practices, and general knowledge of nutrition. The pupils were taught how to recognize the various sources of food and food groups, the names of the different food groups, and how to identify bodybuilding foods, energy-giving foods, and protective foods. They were also educated on the importance of eating from all the food groups. Cards with pictures of different foods from the food groups were used to demonstrate the teaching. To make the teaching practical, participants listed foods they ate the previous day and categorized them into the various food groups. Participants had education on the different types of food nutrients, their sources, and their associated deficiencies. Emphasis was made particularly on specific micronutrients such as vitamins A, D, E, K, B1, B2, iodine, and iron. Demonstrations were done using both cards and PowerPoint Presentations (images and video presentations).

Pupils were assigned into groups and a food-nutrient deficiency game was employed where participants matched foods to images of deficiencies using cards. On healthy eating practices, participants were educated on combining various food groups to obtain a balanced diet. Participants were educated on other nutrition topics, including the need for breakfast consumption, healthy snacking, salt reduction, food safety and hygiene, the effects of hunger, and the need to drink enough water. During demonstrations, the children were assigned to describe how they would eat healthily in a day with a specified amount of money. All questions from the participants were well addressed.

### Component of physical activity education

Participants in this group were educated on various physical exercises and their benefits. They were taught health-related physical fitness and their components, which included muscular endurance, muscular strength, flexibility, and cardiovascular endurance. The participants were engaged in a variety of physical activities during the practical, including a field demonstration of locomotive and non-locomotive skills. Participants were encouraged to participate in sixty (60) minutes of moderate to vigorous physical activities daily, both inside and outside of school hours. At each teaching session, each participant was asked to develop a personal physical activity timetable to be presented on the next visit.

### Combined nutrition and physical activity intervention group

Participants in this group received both nutrition and physical activity education as have been described above. The control group received no intervention.

### Follow up and post-intervention assessment

Follow up data collection was done after three (3) and six (6) months from the start of the intervention. The three months follow-up assessed the impact of a nutrition education intervention on knowledge. The 6 months follow up however involved a repetition of all the baseline assessments: knowledge and BMI-for-age status.

### Data analysis

Data collected were entered into Excel software and analyzed using Statistical Package for Social Science (SPSS version 20, Chicago, IL). Anthropometric data of weight and height were converted to BMI-for-age Z scores (BFA) using WHO Anthro Plus version 10.4. A paired sample T-test was used to assess the overall effect of the intervention, the observed differences between pre-and post-intervention, between intervention and control schools. Repeated measures ANOVA analysis was used to compare the baseline and end of intervention differences of means between the intervention groups and control. General Linear Model for repeated samples and LSD posthoc were performed to compare between-group differences in cognition scores before and after the intervention. All P-values < 0.05 were considered statistically significant.

## Results

**Figure 1:**
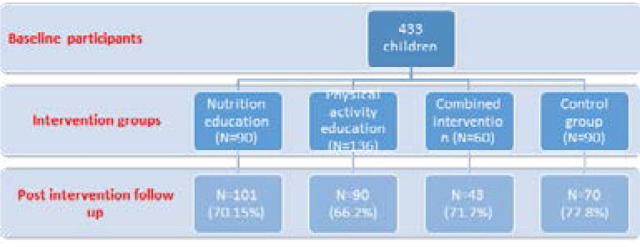
Study flow and design

Figure 1 shows a flow diagram of the study design and procedure. Of the 433 children who were part of the baseline, 304 (70.7%) were available for the end of intervention assessment.

### Effect of intervention on nutrition and physical activity knowledge within the intervention groups

Table 1 shows the mean nutrition and physical activity knowledge performance at baseline and post-intervention between the different groups. Physical activity and nutrition knowledge significantly improved in all intervention groups (P < 0.001), but the magnitude of improvement differed between the different intervention groups. Participants in the nutrition and PA education group improved most by 4.1 points (31.0%), followed by the nutrition education group (3.9 points, 29.8%), with the least improvement in the control group (2.5 points, 19.1%).

**Table 1:** Comparing means nutrition and PA knowledge scores between baseline and postintervention within the different intervention groups

Intervention group		Nutrition and PA knowledge					
			
	Time	N	Means	SD	Mean difference (95%CI)	Percentage gain	P-value
Overall	Baseline	280	12.93	3.426	3.4 (2.9–3.9)	26.3%	<0.001
	Post	280	16.27	3.387			
Control	Baseline	64	13.08	4.029	2.5 (1.5–3.6)	19.1%	<0.001
	Post	64	15.61	3.264			
Nutrition	Baseline	96	13.18	3.350	3.9 (3.1–4.6)	29.8%	<0.001
	Post	96	17.03	3.465			
PA	Baseline	83	12.39	3.238	3.0 (2.3–3.9)	24.2%	<0.001
	Post	83	15.43	3.037			
Both	Baseline	37	13.22	2.849	4.1 (2.9–5.7)	31.0%	<0.001
	Post	37	17.32	3.536			

Data are presented as mean±SD (standard deviation), PA- Physical Activity, P-value is significant at P < 0.05.

### Effect of intervention on nutrition and physical activity knowledge between the intervention groups

Table 2 shows the overall difference between means of nutrition and physical activity knowledge scores of the four intervention groups. At baseline the intervention groups were similar (P = 0.420), meaning their knowledge levels were not different. However, after the intervention, differences existed in knowledge levels between the intervention groups (P < 0.001). Post hoc analysis shows the significant differences of knowledge scores were between the nutrition education group versus the control group (mean change= +1.3, P = 0.007), both intervention group versus the control group (mean change= +1.9, P = 0.006). This was not significant between the physical education group and the control group (-0.1, P = 0.842).

**Table 2:** Mean comparisons and posthoc analysis of Nut and PA knowledge between the different groups

	Control	Nutrition	PA	Both	Treatment effect between control and intervention groups			
	
	Mean±SD	mean±SD	mean±SD	Mean±SD	Mean change(95%CI)	Mean change(95%CI)	Mean change(95%CI)	All
Age	10.8±1.1	11.3±1.1	11.0±1.0	11.3±1.1	Nutrition	PA	Both	
(N)	79	113	104	47				
Baseline K	12.7±3.9	13.0±3.5	12.4±3.3	13.3±2.9				
P value					0.626	0.541	0.337	0.420
N	70	103	92	43				
Endpoint K	15.6±3.2	17.0±3.5	15.4±3.0	17.3±3.3	1.3 (0.3–2.4)	-0.1 (-0.9–1.1)	1.9 (0.6–3.2)	
P value for interaction					0.007	0.842	0.006	<0.001

Data are presented as mean±SD (standard deviation), Post hoc analysis was done using LSD. K-Nutrition and physical activity knowledge, PA- Physical Activity, Both- Nutrition and physical activity, P-value is significant at P < 0.05.

### Effect of intervention on BMI-for-age z-score within the intervention groups

Paired sample ANOVA shows the difference between the baseline and post-intervention BMI-for-age scores was +0.36 showing that BMI-for-age z-scores improved when all participants are lumped together (P < 0.001). Within the intervention types, the mean BMI-for-age score for each intervention group improved and these improvements were all statistically significant (P < 0.05). However, the improvement within the nutrition education group was the highest +0.65 (P < 0.001), compared with +0.23 in the physical activity education group (P < 0.001), +0.27 in nutrition and physical activity group (P < 0.001) with the least in the control group +0.18 (P = 0.001) (Table 3).

**Table 3:** Comparison of baseline and post-intervention BMI-for-age z-scores within intervention groups

Groups		BMI-for-age z-scores					P-value
				
	Time	N	Mean	SD	Mean difference	95%CI of the difference	
Overall	Post	304	0.10	1.16	+0.36	0.28–0.42	<0.001
	Baseline	304	-0.26	0.98			
Control	Post	70	-0.07	1.15	+0.18	0.07–0.28	0.001
	Baseline	70	-0.25	1.12			
Nutrition	Post	101	0.44	1.22	+0.65	0.44–0.79	<0.001
	Baseline	101	-0.21	0.91			
Physical activity	Post	90	-0.08	1.07	+0.23	0.14–0.31	<0.001
	Baseline	90	-0.31	0.95			
Both intervention	Post	43	-0.20	0.98	+0.27	0.16–0.37	<0.001
	Baseline	43	-0.29	1.04			

Data are presented as mean ± SD (standard deviation), CI= Confidence Interval.

P-value is significant at P < 0.05

**Figure 2:**
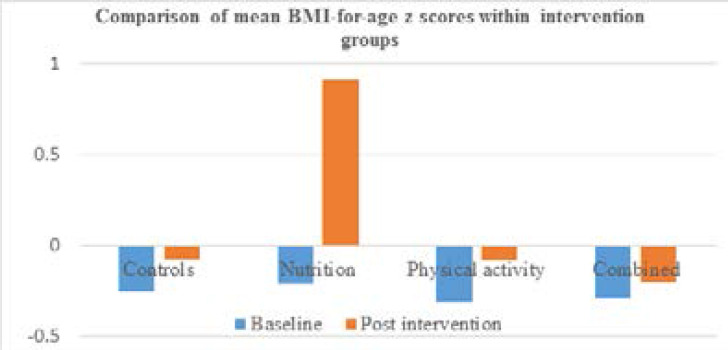
Comparison of mean BMI-for-age z-score within the intervention groups before and after intervention

Figure 2 illustrates the changes in mean BMI-for-age within the different intervention groups following the intervention. Although the mean z-scores were negative at baseline, and these improved in each group, it is only the nutrition group that had a positive BMI-forage mean z-scores after the intervention (Fig. 2).

### Effect of intervention on BMI-for-age z-score between the intervention groups

Table 4 presents the effects of the intervention on BMI-for-age-z-score between the intervention groups. On average, at the end of the intervention, there was an increase in mean BMI-for-age-z-score for all participants (Baseline: -0.26±0.9, endpoint: 0.10±1.1, mean change: 0.4, P<0.001). The mean BMI-for-age-z-score also increased across the groups; control group (mean change: +0.2, P=0.001), nutrition group (mean change: +0.6, P<0.001), physical activity group (mean change: +0.2, P<0.001), both nutrition and physical activity.

**Table 4:** Multiple comparison of BMI-for-age z-scores between intervention groups at baseline and post-intervention

BMI-for-age	All participants	Control	Nutrition	PA	Both	

	Mean	Mean	Mean	Mean	mean	P value
(N for all)	433	91	144	137	61	
Baseline for all	-0.26±0.9	-0.25±1.0	-0.22±0.9	-0.31±0.9	-0.26±1.0	0.904
N	304	70	101	90	43	
Endpoint for all	0.10±1.1	0.07±1.2a	0.4±1.2a,b,c	0.09±1.1b	0.003±1.0c	0.004
Mean change	0.4(0.3–0.4)	0.2(0.1–0.3)	0.6(0.4–0.8)	0.2(0.1–0.3)	0.3(-0.4–0.1)	
N for Girls	223	46	84	64	29	
Baseline BFA Girls	-0.05±0.9	-0.22±1.0	-0.06±0.9	-0.08±0.7	0.05±1.0	0.698
N	160	38	59	41	22	
Endpoint BFA Girls	0.34±1.0	0.11±1.1a	0.63±1.1a	0.15±0.9	0.31	0.053
Mean change	0.5(0.4–0.6)	0.3(0.2–0.4)	0.7(0.4–0.9)	0.3(0.2–0.4)	0.5(0.3–0.6)	
N for Boys	210	45	60	73	32	
Baseline BFA boys	-0.44±1.0	-0.28±1.2	-0.42±0.8	-0.47±1.1	-0.45±1.0	0.834
N	144	32	42	49	21	
Endpoint BFA boys	-0.16±1.2	-0.29±1.2	0.08±1.2	-0.29±1.2	-0.38±1.0	0.190
Mean change	0.2(0.1–0.3)	0.0(-0.1–0.2)	0.5(0.2–0.8)	0.2(0.1–0.3)	0.1(-0.2–0.1)	

Data are presented as mean SD (standard deviation), Same alphabets are significant at P< 0.05, Posthoc analysis showed mean difference for all participants (C vs N, P = 0.004, N vs PA, P= 0.001, N vs B, P= 0.027), and for girls (P = 0.017).

BFA- BMI-for-age, PA- Physical Activity group, C- Control, N- Nutrition group, B- Both intervention group. P value is significant at P < 0.05

Also, the means between the intervention groups when boys (P = 0.834) and girls (P = 0.690) are analyzed separately are not significantly different. However, post-intervention differences in BFA are significantly different for overall participants (P = 0.004), and for girls (P = 0.050) but not for boys (P = 0.190). Between the intervention groups, the BFA after the intervention between the nutrition education group and control group (P = 0.004), nutrition education group and PA education group (P = 0.001), and nutrition education group and both intervention group (P = 0.020) are different, while the rest, control group versus PA education group, control group versus both intervention group and PA education group versus both intervention group are not different. Comparatively, the nutrition education group's mean BMI-for-age z-scores was significantly higher than the controls level by +0.51 (Mean BMI-for-age; Nutrition education group: 0.44; control group: -0,07, (P = 0.004), and between the nutrition education group and PA education group (mean difference: +0.53, mean BMI-for-age; Nutrition education group: 0.44; PA education group: -0.09, P = 0.001) but not between any of the other groups (P > 0.050).

When the group is split by pupils with BFA z-score less than -1 at baseline and those equal or above -1 SD at baseline, it is observed that initial mean BFAs between the four intervention groups are not different at baseline. After the intervention, however, among those that had BFA <-0.1 SD, the nutrition education group versus the control group are different (P = 0.021), the nutrition education group and PA education group different (P = 0.002), and the nutrition education group and both groups nearly different (P = 0.050). Also, among those with BFA z-score equal or above -0.1 SD, the intervention groups are different after the intervention (P = 0.040), and the differences are only significant between the nutrition education goup and controls group (P = 0.040), and both group and PA education group (P = 0.010) (Table 4).

**Figure 3:**
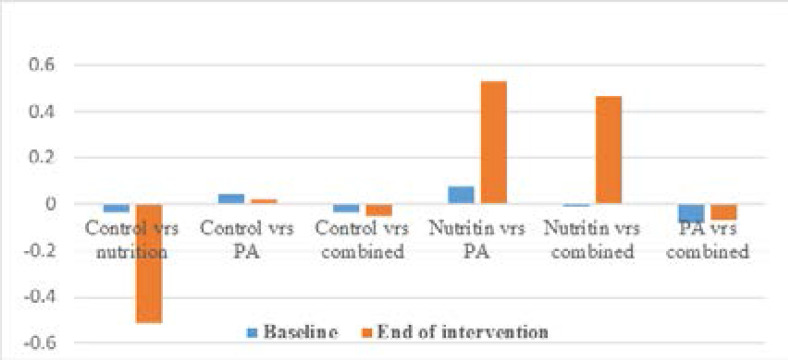
Comparing mean BFA z-scores between the intervention groups at baseline and post-intervention

The BMI-for-age z-scores between the intervention groups are compared further in Figure 3. As shown, the control group was lower than the nutrition education group in BMI-for-age z-score by a mean of -0.03 and this gap widened to -0.51 (first orange bar to the left). Likewise, the nutrition education group and PA education group (mean difference at baseline +0.07 versus +0.53 end of the intervention (fourth orange bar from left), and the nutrition education group and nutrition and physical activity education group groups (mean difference at baseline -0.001 versus +0.46 end of intervention) were much closer at baseline but the gaps had widened following the intervention, showing that the nutrition education group improved better than all the different groups (Fig 3).

### Associations between baseline and post-intervention knowledge and BFA z-scores

**Figure 4:**
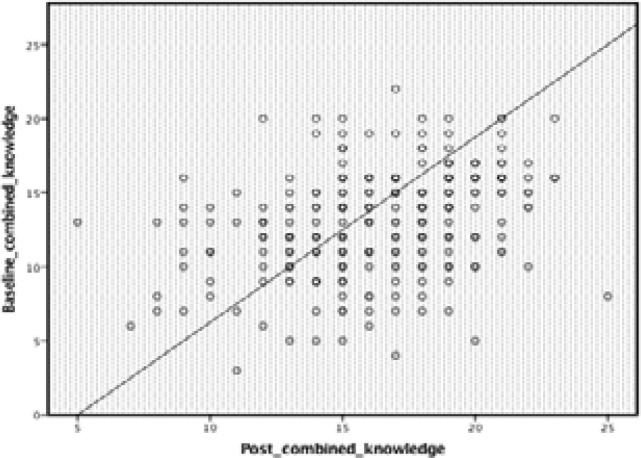
Scatter plot of association between baseline and post intervention nutrition and PA knowledge score

**Figure 5:**
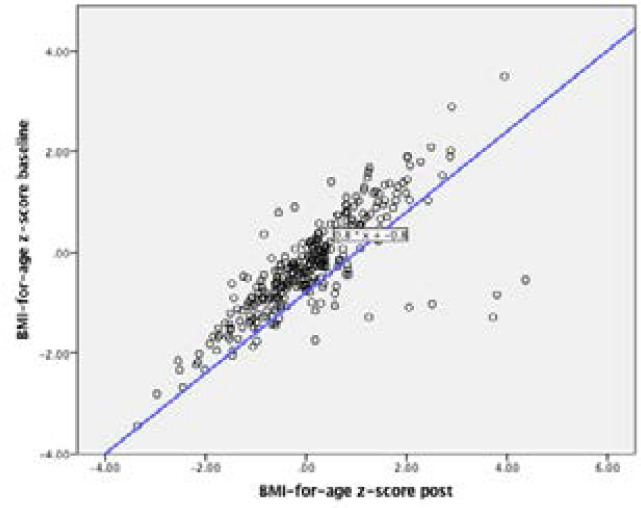
Scatter plot of association between baseline and post-intervention BFA z-score

Scatter plots illustration in Figures 4 and 5 together with Table 5 show a strong linear positive correlation between baseline and end of intervention physical activity and nutrition knowledge, indicating that the better the knowledge at baseline and better the knowledge after the intervention.

**Table 5:** Correlation coefficients and statistical significance between baseline and post-intervention knowledge and BFA z-scores

	Post total knowledge	Baseline BFA	Post BFA
	
	Correlation coefficient (r)		
Baseline total knowledge	0.331**	-0.016	0.006
Post nutrition knowledge	0.921**	-0.011	0.015
Post PA knowledge	0.688**	-0.047	-0.089
Post total knowledge		-0.03	-0.029
Baseline BFA			0.756**

BFA- BMI-for-age, Correlation coefficients with 2 asterisks are significant at p < 0.001 while those without any asterisks are of p > 0.05. Partial correlations controlled for age, gender and type of intervention

## Discussion

The study evaluated the effectiveness of physical activity and nutrition education on nutrition knowledge and BMI-for-age among school-aged children aged 9–13 years old, attending government-owned basic schools. The average age between the children in the four different groups was similar, indicating that groups were of similar age. Among children, nutrition education is important in improving growth and preventing undernutrition because it can promote healthy dietary habits and food choices.^28^ Non-Communicable diseases (NCDs) are highly prevalent among adults in sub-Saharan African countries and is steadily increasing in children.^29^ On the other hand, undernutrition has dire consequences and is still prevalent in children.^30^–^32^ The school-aged population, however, is the neglected group with regards to nutrition interventions to improve BMI status, warranting the present study.

Our findings revealed that nutrition and physical activity knowledge did not differ between groups at baseline. However, physical activity and nutrition knowledge significantly improved in all groups and between groups (control groups versus nutrition group, and control group vs both nutrition and physical activity group), at the end of the study. The treatment effect between the control and the intervention showed that nutrition and physical activity knowledge had a positive effect (mean change) for the nutrition group (+1.3, P= 0.007), and both the nutrition and physical activity group (+1.9, P =0.006), but a negative effect for physical activity group (-0.1, P = 0.842). This implies that compared to the control group, the nutrition and physical activity education given to the children improved participants in the nutrition group, and both nutrition and physical activity groups. However, the improvement in nutrition and physical activity knowledge was observed across the groups at the end of the intervention. The study used the same knowledge test at baseline and post-intervention; hence, it is expected that familiarity with the test questions could have resulted in some improvement across the groups. Other studies have reported cross-communication between the studied group as a limitation in this type of study.^16^,^17^. Also, the study did not assess their curriculum to know if nutrition and physical education lessons were taught in a particular selected school. This could also influence the results. Despite familiarity with test questions, mean change (confidence intervals) of physical and nutrition knowledge scores varied between intervention groups at the end of the study. The participants in the nutrition and physical activity group (+4.1) had the most improved knowledge, followed by the nutrition group (+3.9), PA education group (+3.0), and the control group (+2.5), having the least improvement in knowledge. This means that nutrition and physical activity education was an effective intervention in improving the physical activity and nutrition knowledge of school children, and those that received education on both nutrition and physical improved most in knowledge. A study by Kostanjevec et al.33, evaluating the effectiveness of nutrition education on nutrition knowledge of grade 6 elementary school children in Slovenia found that formal nutrition education improved the nutrition knowledge of participants.

The effect of the interventions on nutritional status was assessed using BMI-for-age z-score. The BMI-forage index is used for monitoring the growth of schoolaged children and adolescents^26^. When participants were put together, there was a significant difference in mean BMI-for-age z scores between baseline and post-intervention (P < 0.001). An improved BMI-for age was observed post-intervention across the groups, and this could partly be a result of the nutrition and physical activity education received. Across the group, the improvement in BMI-for-age was seen highest in the nutrition education group (+0.65), followed by both nutrition and physical activity group (+0.27), physical activity group (+0.23), and the least; control group (+0.18). However, this improvement also occurred in the control group who had no nutrition and physical activity intervention since the study did not influence the study environment; activities engaged by the participants during playing times at school, after school, and days there were no nutrition and physical activity classes. We cannot also tell if some of these participants in the control schools and intervention schools lived together in the communities; in which there is likely cross-communication of knowledge received by the intervention groups to the control groups. Also, the study had no influence on their dietary intake and physical activities engaged by both groups at home. Some studies have reported that nutrition education intervention among school children is limited to cross-communication of nutrition and physical activity knowledge received between the intervention group the control group, and self-reporting of study variables such as dietary behaviour and physical activity level^16^, ^17^. A posthoc analysis revealed a significant difference between the groups., and participants in the nutrition education group had the highest significant change in BMI-for-age. The mean BMI-for-age (BFA) improved among all participants by +0.36. Additionally, mean BFA did not differ between groups at baseline and when the 4 intervention groups were stratified by gender and compared, they were not different at baseline. The mean BFA differed significantly between groups for the girls' comparison but not the boys. A posthoc analysis revealed that the nutrition group had the largest improvement in BFA by comparison of girls, compared to the PA group, nutrition, and physical activity group, and control group. So clearly, the intervention improved BFA in those who received the nutrition education most, and better than the other intervention groups.

A study by Tilles-Tirkkonen et al.^22^ concluded that nutrition education is a powerful tool in promoting health as well as improvement in the eating habits of children. One nutrition education study by Addo et al.^25^ which used 54 school children at Ga-East Municipality in Accra found that short-term nutrition education using posters, flyers, and practical sessions improved nutrition knowledge and lipid profile of overweight/obese school children. Another study by Briggs et al. 34 that examined the efficacy of nutrition education curriculum to be used by teachers reported that fifth graders in the intervention school improved their breakfast frequency, increased their consumption of vegetables, and reduced their consumption of ice cream, sweets, and sugar-sweetened drinks while no improvement was found in the fifth graders at the control schools. 34 This study also involved fifth graders and although the effect of the intervention on dietary intake was not our focused, the assumption from this study is that education led to improved knowledge, which led to better dietary practices, which reflected in anthropometric status, that is BMI-for-age.

It is reported that obesity in childhood particularly adolescence is a key predictor of obesity in adulthood^35^. We observed strong positive correlations between baseline and end of intervention nutrition knowledge, and between baseline and end of intervention BFA, signifying that the higher the knowledge and BFA at baseline, the higher they were after the intervention. This may have a negative connotation since those on the higher side of BFA were likely to become more overweight/obese. The study implies that nutrition education to some extend can be used as a low-cost effective intervention program to improve knowledge and anthropometric variables of school children in a well-controlled study design. As stated above, the study is limited to some factors. The study did not assess variation in activities undertaken in each school environment, and the study did not assess the nutrition lessons taught in each school as well as the physical activity level of the participants. Te study did not assess whether some schoolchildren were absent and had lapsed during the time of the intervention. The findings therefore should be interpreted with caution.

## Conclusions and recommendations

At the end of the study, nutritional and physical activity knowledge, and BMI-for-age improved significantly among the school-aged children receiving nutrition and physical activity education intervention compared to controls. The school-aged children receiving both nutrition and physical activity intervention gained the most in knowledge, followed by the nutrition intervention group. Also, at the end of the study, children receiving nutrition intervention had the largest improvement in anthropometric status (BMI-for-age), followed by both intervention groups. Thus, education had a positive impact on the knowledge and BMI status of the children and would be a cost-effective strategy for lasting impact on the nutritional status of school-aged children. However, further studies on the long-term impact of nutrition education in other regions of the country and within and between public and private schools are recommended to provide more nationally-representative data. This will inform nutrition policies that target this age group.
